# A perspective on the fundamental theory of nonaqueous electrolytes for rechargeable batteries

**DOI:** 10.1093/nsr/nwae394

**Published:** 2024-11-05

**Authors:** Xiang Chen, Nan Yao, Zhao Zheng, Yu-Chen Gao, Qiang Zhang

**Affiliations:** Tsinghua Center for Green Chemical Engineering Electrification (CCEE), Beijing Key Laboratory of Green Chemical Reaction Engineering and Technology, Department of Chemical Engineering, Tsinghua University, China; Tsinghua Center for Green Chemical Engineering Electrification (CCEE), Beijing Key Laboratory of Green Chemical Reaction Engineering and Technology, Department of Chemical Engineering, Tsinghua University, China; Tsinghua Center for Green Chemical Engineering Electrification (CCEE), Beijing Key Laboratory of Green Chemical Reaction Engineering and Technology, Department of Chemical Engineering, Tsinghua University, China; Tsinghua Center for Green Chemical Engineering Electrification (CCEE), Beijing Key Laboratory of Green Chemical Reaction Engineering and Technology, Department of Chemical Engineering, Tsinghua University, China; Tsinghua Center for Green Chemical Engineering Electrification (CCEE), Beijing Key Laboratory of Green Chemical Reaction Engineering and Technology, Department of Chemical Engineering, Tsinghua University, China

## Abstract

The chemical origins of the major differences between nonaqueous electrolytes and classical solution models were delved to promote the future development of battery electrolyte theories.

The electrolyte, which mainly conducts ions and insulates electrons between electrodes, is one of the most important parts of rechargeable batteries and significantly influences battery performance [1]. The working temperature of a battery is directly related to the melting point and boiling point of electrolytes. A high flash point is supposed to improve battery safety. A low viscosity and a large ionic conductivity are prerequisites for electrolytes. Besides, routine electrolytes are intrinsically unstable against low-potential anodes and high-potential cathodes, leading to the formation of a solid electrolyte interphase (SEI) between the electrolyte and the electrode. The components and physicochemical properties of SEI are highly dependent on electrolyte compounds, which obviously influence the electrochemical performances of batteries.

**Figure 1.**
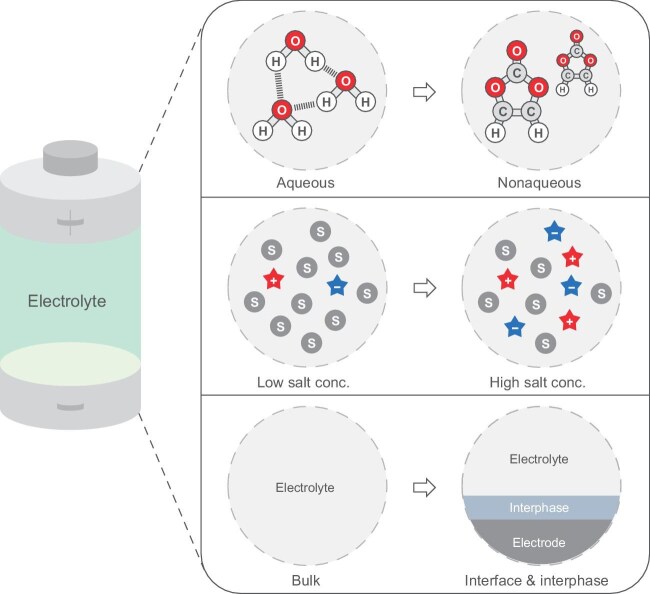
Schematic representation of the origin of the major differences between lithium battery electrolyte chemistry and classical solution chemistry. conc., concentration.

Electrolytes can be generally classified into aqueous electrolytes and nonaqueous electrolytes. Despite the great achievements in aqueous electrolytes, nonaqueous electrolytes are the most widely applied systems due to their competitive advantages, especially in commercialized lithium ion batteries [2]. However, classical solution theories predominantly focus on aqueous solutions, and the fundamental theory for nonaqueous electrolytes is relatively lacking, which impedes the exploration of battery electrolyte chemistry and the rational design of advanced electrolytes.

In this perspective, we aim to provide a comprehensive and in-depth understanding of the fundamental differences between nonaqueous electrolytes in batteries and aqueous electrolytes in classical solution models, and further afford a fruitful insight into the future development of battery electrolyte theories (Fig. 1).

**(1) From aqueous electrolyte to nonaqueous electrolyte.** Water molecules, which are both electrophilic and nucleophilic, are the major components of aqueous electrolytes. As a result, both cations and anions in aqueous electrolytes can form relatively strong interactions with water molecules, resulting in a typical solvation structure. However, electrophilic solvents represented by water molecules often possess strong acidity and are easily reduced on the anode surface, producing flammable hydrogen gas. In contrast, organic solvents used in rechargeable batteries are usually only nucleophilic, which induces a fragile interaction between anions and solvents. Besides, organic solvents generally possess a smaller dielectric constant than water (78.3 at 25^o^C), indicating a weaker shielding effect of the electrostatic interactions between cations and anions. Therefore, anions in nonaqueous electrolytes are mainly present as free ions, contact ion pairs, and aggregates. Last but not least, there is only one aqueous electrolyte solvent namely water but there are many choices of organic solvents. Mixed solvents such as ethylene carbonate/diethyl carbonate and 1,3-dioxolane/1,2-dimethoxyethane are widely adopted for battery electrolytes to maximize the advantages of various molecules while simultaneously minimizing their disadvantages. It should be noticed that the mixing effects of different solvent systems can be very different [3].

According to the theoretical model of ion–solvent complexes, molecular interactions in electrolytes significantly influence the electronic structure and electrochemical stability of electrolyte species [4–6]. For example, cations can reduce the energy level of the lowest unoccupied molecular orbital (LUMO) of solvents, and the change in the LUMO energy level is strongly related to the binding energy between the cation and the solvent. A lower LUMO level indicates the molecule can more readily obtain electrons from the anode in principle. In reverse, anions are expected to increase the LUMO energy level of solvents and thus reductive stability [5]. However, the interaction between anions and solvents is often very weak and the influence of anions in solvents is not pronounced. In practice, anions can participate in the solvation shell of Li^+^, which results in the decreased and increased LUMO energy level of the anion and solvent in the solvation shell, respectively. Despite that a linear relationship was also observed for the change of LUMO energy level and reductive potential of anions, the gap between the LUMO energy and reductive potential should be noticed.

Besides the first solvation shell, the second solvation shell structures are also very different in aqueous and nonaqueous electrolytes. Hydrogen bonds can be formed between water molecules, which can induce a localized ordered structure due to the directionality of hydrogen bonds. The directly arranged water molecules in the first solvation shell can regulate the orientation of water molecules in the second solvation shell through the hydrogen bonds, inducing a localized ordered solvation structure. However, hydrogen bonds can hardly be observed in nonaqueous electrolytes due to the lack of a hydrogen donor, resulting in a relatively weak interaction between organic solvents. Although the concept of the second solvation shell is widely applied to nonaqueous electrolytes, its characteristics are not as typical as those in aqueous electrolytes [7].

A common characteristic of aqueous and nonaqueous electrolytes is that solvents can decompose on electrode surfaces and produce SEI, especially when the electrode potential is very high or low. However, the decomposition products of organic solvents are very different from water molecules. Organic solvents in nonaqueous electrolytes can decompose and produce both organic components, such as ROCO_2_Li, ROLi, and polymer, and inorganic components, such as Li_2_CO_3_, LiF, and Li_2_O. For example, some organic solvents such as 1,3-dioxolane and trioxane can be polymerized to improve the mechanical properties of SEI. The inorganic SEI components mainly act as a barrier to inhibit the direct contact between the electrolyte and the anode, further restricting the consumption of electrolytes. Consequently, regulating the ratio and spatial distribution of organic and inorganic compounds in SEI by electrolyte design is an important and practical strategy for building a stable electrolyte–electrode interface in rechargeable batteries.

**(2) From low-concentration electrolyte to high-concentration electrolyte.** In classical solution models, strong and weak electrolytes are defined to differentiate the dissociation behaviors of solutes in solvents. Most salts tend to dissociate in water due to water's strong polarization and produce a strong electrolyte, in which ions are mainly solvated by water molecules. However, the above principle can only be applied to dilute solutions, and salt concentration can significantly change electrolyte solvation structures. As the salt concentration increases, the solvent-to-ion ratio decreases, and ions cannot be fully coordinated by solvents. As a result, more ion contact pairs and even aggregates will be formed at a higher salt concentration.

For commercialized lithium ion batteries, 1.0 M LiPF_6_ electrolyte with promising ionic conductivity is widely adopted, and the concentration is much higher than the scope of applying the Debye–Hückel equation (<0.01 mol kg^−1^). On the one hand, the salt concentration of 1.0 M is far from ideal in dilute electrolytes. On the other hand, anions are easier to participate in the cation solvation shells in nonaqueous electrolytes due to the lower polarization of organic solvents compared with water. Namely, the critical salt concentration when the anions participate in the solvation shell of cations is much lower for nonaqueous electrolytes than for aqueous electrolytes. More extremely, some salts such as lithium polysulfides can hardly disassociate in nonaqueous electrolytes and exhibit a very complicated equilibrium [8]. Therefore, classical dilute solution models cannot be directly applied to battery electrolytes, and the negligible cation–anion interactions should be considered in future model improvements.

Besides solvation structures, the salt concentration has a significant influence on the physicochemical properties of electrolytes, including dielectric constant, viscosity, and ion transport properties. For example, the dielectric constant increases first and then decreases as the salt concentration increases [3]. At relatively low salt concentrations, strong dipoles of ion contact pairs will increase the dielectric constant due to their large dipole moments. However, the movement of electrolyte species will be resisted at relatively large salt concentrations due to the formation of large solvation clusters, resulting in a decreased dielectric constant. The large clusters connected by anions also deliver a strong interaction between electrolyte species, which further decreases the diffusion coefficient of solvents and increases the electrolyte viscosity [9]. The correlation between viscosity and electrolyte species interaction can be formulated by an exponential function,


\begin{eqnarray*}
\eta = {\mathrm{A}}{{e}^{ - \frac{{{{E}_{\mathrm{b}}}}}{{\alpha {{k}_{\mathrm{B}}}T}}}},
\end{eqnarray*}

where A and α are pre-exponential and exponent fitting coefficients, respectively, *T* the temperature, and *E*_b_ the binding energy between electrolyte species.

**(3) From bulk electrolyte to interface and interphase.** The model of electric double layer (EDL) was proposed to explain the difference between the bulk electrolyte and the electrolyte at the electrode interface. The classical EDL includes the ions adsorbed on the electrode surface (Stern layer) and a film of the countercharged dispersion medium (diffuse layer). The electrolyte structures in EDL are distinct from bulk electrolytes, especially for the inner layer on highly charged electrodes. For example, an electrode with a very low potential is supposed to enrich the negative charge and further preferentially attract cations in electrolytes. The unique structure in EDL further changes the potential distribution and ion transport properties. However, the influence of SEI on EDL is not considered in classical models. First, the SEI, especially organic components, possesses a complicated porous structure and can hold a specific amount of electrolytes. Second, the thickness of SEI is comparable to that of EDL. The concentration gradient distribution is different above the electrode surface and the SEI surface. Third, the potential decrease mainly exists in the SEI region, namely the potential at the SEI surface is comparable to that of bulk electrolytes. All these factors weaken the influence of the electrode potential and electrode properties in the electrolyte structure above the SEI surface. The EDL structure above SEI is largely determined by SEI itself and the bulk electrolyte, which is different from classical models.

Designing advanced nonaqueous electrolytes is one of the most promising and practical approaches for promoting the next-generation rechargeable batteries. However, classical solution models face many limitations in guiding rational electrolyte design. This perspective summarizes the major gap between nonaqueous electrolytes and model solutions, including influences arising from nonaqueous solvents with only nucleophilicity, salt concentrations, and SEI. To narrow these gaps, the following directions can be considered in the future.

**(1) Atomic simulation of both bulk electrolyte and EDL.** With the rapid development of computer science, atomic simulations are strongly supposed to probe the electrolyte solvation structure and corresponding properties. Especially, the combination of machine learning techniques promotes the simulation of a large model with high accuracy; enhanced sampling methods such as the splitting–overlapping–screening (SOS) method [9] can significantly improve the prediction accuracy of electrolyte properties such as viscosity; constant potential methods can reveal the mysterious structure of EDL even considering complicated electrode structures.

**(2) Advanced characterizations for battery electrolytes.** Apart from the information on free ions, contact ion pairs, and aggregates in electrolytes, many structural parameters, such as shape, particle size, and internal structure over multiple spatiotemporal scales, are urgently required to reveal electrolyte structure–function relationships. Advanced characterizations such as X-ray/neutron scattering, infrared/Raman spectroscopy, and nuclear magnetic resonance spectroscopy can provide structural information from sub-nm to μm scale. Meanwhile, developing *operando* scattering techniques with high time resolution and a suitable sample environment can help uncover the dynamic structural evolution behavior of electrolytes under working conditions.

**(3) Artificial intelligence for electrolyte (AI4Electrolyte).** The artificial intelligence approach is strongly supposed to reconstruct scientific discovery by empowering current theoretical and experimental tools from the microscale to the macroscale [10]. Regarding battery electrolytes, AI4Electrolyte can unveil the complicated solvation structure at the interface and (de)solvation process by integrating advanced characterizations, multiscale simulations, and machine learning methods. The underlying chemical principles can be further discovered based on data-driven approaches such as symbolic learning. Novel electrolyte molecules can be designed in billions of molecular spaces by combining generative methods and high-throughput screening. For example, electrolyte properties such as dielectric constant, viscosity, ionic conductivity, binding energy with a Li ion, geometric and electronic structures, and formation energy can be adopted as screening descriptors. Besides, electrolyte recipes can be quickly optimized through active learning and high-throughput experiments such as chemical robots.
